# Medicaid Coverage in Early Childhood for Children With Sickle Cell Disease

**DOI:** 10.1001/jamanetworkopen.2024.21491

**Published:** 2024-07-12

**Authors:** Sophia S. Horiuchi, Sarah L. Reeves, Allison P. Plaxco, Hannah K. Peng, Mei Zhou, Mariam Kayle, Mary Hulihan

**Affiliations:** 1 Tracking California Program, Public Health Institute, Oakland; 2 Susan B. Meister Child Health Evaluation and Research Center, Medical School, Department of Pediatrics, University of Michigan, Ann Arbor; 3 Division of Epidemiology, Biostatistics, and Environmental Health, School of Public Health, University of Memphis, Memphis, Tennessee; 4 Georgia Health Policy Center, Andrew Young School of Policy Studies, Georgia State University, Atlanta; 5 Duke University School of Nursing, Durham, North Carolina; 6 Division of Blood Disorders and Public Health Genomics, National Center on Birth Defects and Developmental Disabilities, Centers for Disease Control and Prevention, Atlanta, Georgia

## Abstract

This cohort study examines patterns of Medicaid coverage in the first 3 years of life among children with sickle cell disease across 5 states.

## Introduction

Infants born in the US are screened for sickle cell disease (SCD) through state-based newborn screening (NBS) programs.^1^ Universal NBS and improvements in preventive care have led to improved survival rates for individuals with SCD.^2^ However, gaps in access to high-quality health care, due, in part, to the persistence of structural and interpersonal racism, have resulted in children receiving inadequate care for the disease.^3,4,5^ Our objective was to examine patterns of Medicaid coverage among children with SCD in the first 3 years of life, a critical period to improve access to high-quality pediatric care.

## Methods

This cohort study included newborns identified from 2015 to 2017 in 5 states—California (CA), Georgia (GA), Michigan (MI), North Carolina (NC), and Tennessee (TN)—and determined to have SCD by their state’s NBS program. These states are in the Sickle Cell Data Collection program, a population-wide public health surveillance system for SCD funded by the Centers for Disease Control and Prevention. Within each state, NBS records were linked to Medicaid enrollment data to assess monthly Medicaid enrollment status, starting at birth month and continuing for 36 calendar months. For those ever insured by Medicaid within the first 3 years, coverage patterns were evaluated using age at first enrollment, total number of months of enrollment, and interruptions or loss of coverage. The institutional review boards of the California Committee for the Protection of Human Subjects, Georgia State University, Duke, University of Michigan, and the Tennessee Department of Health considered this study exempt, and informed consent was not required because it was secondary analysis of data collected as part of public health surveillance. We followed the STROBE reporting guideline and used SAS, version 9.4 (SAS Institute) for all analyses.

## Results

A total of 1273 newborns with SCD were reported by state NBS programs between 2015 and 2017 (246 in CA, 471 in GA, 184 in MI, 233 in NC, and 139 in TN); overall, 652 (51.2%) were male. Within the first 3 years of life, 1027 (80.7%) were enrolled in Medicaid at some point (Table). For children with any Medicaid coverage in their first 3 years, the median age of first enrollment was 2 months (IQR, 0-8 months) for CA and 0 months (IQR, 0-0 months) for GA, MI, NC, and TN. Once children were enrolled, the median total coverage was 33 months for CA and 37 months for GA, MI, NC, and TN. Overall, 312 children (30.4%) enrolled in Medicaid had interruptions or loss in coverage (Figure).

**Table.** Medicaid Enrollment Patterns for Children With Sickle Cell Disease From Birth to 3 Years of Age

State	Study population, No.	Ever covered by Medicaid, No. (%)	Ever covered by Medicaid, median (IQR), mo	
			Age at first enrollment	Total Medicaid enrollment
California	246	162 (65.9)	2 (0-8)	33 (24-37)
Georgia	471	380 (80.7)	0 (0-0)	37 (32-37)
Michigan	184	173 (94.0)	0 (0-0)	37 (30-37)
North Carolina	233	197 (84.5)	0 (0-0)	37 (37-37)
Tennessee	139	115 (82.7)	0 (0-0)	37 (37-37)
Overall	1273	1027 (80.7)	NA	NA

Abbreviation: NA, not applicable.

**Figure.**
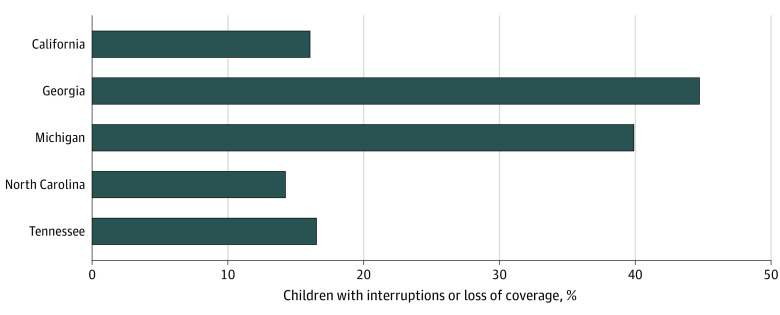
Percentage of Children With Sickle Cell Disease With Interruptions or Loss of Medicaid Coverage Between Initial Enrollment and 3 Years of Age

## Discussion

Given the high lifetime morbidity and disease-related complications associated with SCD, early detection by NBS and subsequent enrollment into health insurance coverage are a public health priority. Our findings indicate that, across 5 states, Medicaid insured more than 80% of children living with SCD for 1 or more months in the first 3 years of life. These results emphasize that Medicaid programs are uniquely positioned to enhance early care initiation, ensure consistent coverage, and implement recommended preventive services to improve health outcomes of the pediatric SCD population. Compared with commercial insurance, state Medicaid programs tend to cover people with more severe SCD-related outcomes resulting in higher overall costs of care.^6^ Therefore, improving delivery and quality of health care beginning early in life for those with SCD is an important consideration for Medicaid programs and works toward decreasing long-term morbidity and costs of care.

Medicaid enrollment outside the birth state was not captured in this study. Differences observed across states could be caused by variations in administrative practices. For example, the higher median age at first enrollment in CA (2 months) may be because newborns are assigned their mother’s Medicaid number for the first 60 days.

In this study, early identification of newborns with SCD through states’ NBS programs was noted as an opportunity for Medicaid programs to leverage their policies and systems to improve the receipt of peventive services and access to specialized care for these children, particularly in the advent of curative therapies for this population.

## Notes

Supplement.
Data Sharing Statement
