# Supplementary material for: Medicaid Coverage in Early Childhood for Children With Sickle Cell Disease
Source: JAMA Netw Open. 2024 Jul 12;7(7):e2421491. doi: 10.1001/jamanetworkopen.2024.21491 (PMC11245719; doi:10.1001/jamanetworkopen.2024.21491)
Supplement: Supplement. — Data Sharing Statement [file jamanetwopen-e2421491-s001.pdf]

Data Sharing Statement

Horiuchi. Medicaid Coverage in Early Childhood for Children With Sickle Cell Disease. *JAMA Netw Open*. Published July 12, 2024. doi:10.1001/jamanetworkopen.2024.21491

Data
Data available: No

Additional Information
Explanation for why data not available: Data used in this paper are housed within individual state SCDC programs under state specific Data Use Agreements. Data are not publically available.
